# Surveillance and Testing for Middle East Respiratory Syndrome Coronavirus, Saudi Arabia, March 2016–March 2019

**DOI:** 10.3201/eid2607.200437

**Published:** 2020-07

**Authors:** Abdullah Alzahrani, Stephanie A. Kujawski, Glen R. Abedi, Safaa Tunkar, Holly M. Biggs, Nada Alghawi, Hani Jokhdar, Abdullah M. Assiri, John T. Watson

**Affiliations:** Ministry of Health, Riyadh, Saudi Arabia (A. Alzahrani, S. Tunkar, N. Alghawi, H. Jokhdar, A.M. Assiri);; Centers for Disease Control and Prevention, Atlanta, GA, USA (S.A. Kujawski, G.R. Abedi, H.M. Biggs, J.T. Watson)

**Keywords:** Middle East respiratory syndrome coronavirus, MERS-CoV, Saudi Arabia, surveillance, viruses, coronavirus, respiratory infections, zoonoses, testing

## Abstract

During March 2016–March 2019, a total of 200,936 suspected cases of Middle East respiratory syndrome coronavirus infection were identified in Saudi Arabia; infections were confirmed in 698 cases (0.3% [0.7/100,000 population per year]). Continued surveillance is necessary for early case detection and timely infection control response.

Middle East respiratory syndrome coronavirus (MERS-CoV) can cause severe respiratory illness and has a reported case-fatality rate of ≈35% (*1*). Transmission typically occurs through close contact with MERS-CoV–infected patients, particularly in healthcare settings (*2*,*3*), or through contact with dromedaries (*4*). Most cases worldwide have been reported by Saudi Arabia (*1*).

By using the Health Electronic Surveillance Network (HESN), a national electronic surveillance platform, the Saudi Arabia Ministry of Health (MoH) monitors MERS-CoV testing and cases throughout the country. Since 2015, clinicians and health authorities have been mandated to report all suspected MERS-CoV cases to HESN. In April 2018, the MoH revised the case definition for suspected MERS-CoV (*5*). This change provided an opportunity to assess testing practices under 2 different case definitions. We describe trends in MERS-CoV surveillance and laboratory testing in Saudi Arabia during March 1, 2016–March 20, 2019.

## The Study

In Saudi Arabia, persons meeting the MoH case definition for suspected MERS-CoV infection are tested for the virus (Table 1) (*5*,*6*). Persons may also be tested if recommended by an infectious disease consultant or if they had exposure to a MERS-CoV patient (*5*,*6*). In April 2018, the MoH published a 4-category revision to the 2015 case definition, with the goal of making the definition more specific (Table 1) (*5*,*6*). This revision was implemented in HESN in July 2018.

**Table 1:** Case definitions for suspected MERS-CoV infection, Ministry of Health, Saudi Arabia, 2015 and 2018*

2015 case definition	2018 case definition
Acute respiratory illness with clinical and/or radiologic evidence of pulmonary parenchymal disease (pneumonia or ARDS)	Severe pneumonia (severity score >3 points) or ARDS (based on clinical or radiologic evidence)
A hospitalized patient with healthcare-associated pneumonia based on clinical or radiologic evidence	Unexplained deterioration of a chronic condition of patients with congestive heart failure or chronic kidney disease on hemodialysis
Upper or lower respiratory illness within 2 weeks after exposure to a confirmed or probable case of MERS-CoV infection	Acute febrile illness (temperature >38°C) with or without respiratory symptoms AND epidemiologic link†,‡
Unexplained acute febrile illness (temperature >38°C) AND body aches, headache, diarrhea, or nausea/vomiting, with or without respiratory symptoms, AND leucopenia (white blood cells <3.5 × 10^9^/L) and thrombocytopenia (platelets <150 × 10^9^/L)	Gastrointestinal symptoms (diarrhea or vomiting) AND leucopenia (white blood cells <3.5 × 10^9^/L) or thrombocytopenia (platelets <150 × 10^9^/L) AND epidemiologic link†,‡
unexplained severe pneumonia or meets >1 of the above case definition categories and in 14 days prior to symptom onset, has exposure to camels or camel products or to a confirmed or suspected MERS-CoV case-patient.	

*Case definitions apply to adults only (>14 years of age) unless indicated. ARDS, acute respiratory distress syndrome; MERS-CoV, Middle East respiratory syndrome. †Applies to children and adults. ‡Epidemiologic link defined as, within 14 days before symptom onset, exposure to a person with a confirmed case of MERS-CoV infection, visit to a healthcare facility where any MERS-CoV patient has been identified or treated within the preceding 2 weeks, or contact with dromedary camels or consumption of camel products.

Information on suspected and confirmed cases is submitted electronically to HESN. For each suspected MERS-CoV case, the treating hospital creates an electronic record in HESN and submits a clinical sample to a designated laboratory for confirmatory testing. After testing is complete, the laboratory updates the electronic record in HESN with the results. Upon report of a confirmed case, additional clinical and epidemiologic data are entered into the HESN system by the hospital, local health authorities, or both.

We analyzed demographic and laboratory data for suspected and confirmed MERS-CoV cases reported to HESN during March 1, 2016–March 20, 2019 by using Microsoft Excel (https://www.microsoft.com) and SAS 9.4 (https://www.sas.com). For this analysis, we defined a suspected case as suspected MERS-CoV infection in a person with compatible symptoms during the study period, with >14 days separating illness episodes for persons in whom MERS-CoV infection is suspected more than once. We defined a confirmed case as laboratory confirmation of MERS-CoV infection in a person during the study period. By using MoH population estimates (*7*), we calculated rates of testing and positivity for the country, by local Health Affairs Directorate (HAD), and for Hajj pilgrims.

During the study period, 200,936 suspected MERS-CoV case-patients were tested and their cases reported to HESN; MERS-CoV was confirmed in 698 case-patients (0.3%) (Table 2). Overall, 54.3% of suspected case-patients were male, 72.8% were Saudi nationals, and the median age was 47 years (interquartile range 28–67 years). Among suspected case-patients for whom healthcare personnel status was available, 5.6% (n = 9,222) were healthcare personnel. Among confirmed case-patients, 517 (74.1%) were male, 501 (71.8%) were Saudi nationals, and the median age was 54 years (interquartile range 40–65 years). The age group with the highest proportion of confirmed case-patients was 50–65 years (0.6%), and the group with the lowest proportion was 0–14 years (0.02%). Healthcare personnel status was reported for 598 (85.7%) confirmed case-patients; among these, 11.2% (n = 67) were healthcare personnel. Outcome information was available for 84.0% (n = 586) of the confirmed case-patients, 164 (28.0%) of whom died.

**Table 2:** Demographic characteristics of persons with suspected and confirmed MERS-CoV infection, Health Electronic Surveillance Network, Saudi Arabia, March 1, 2016–March 20, 2019*

Characteristic	Total	Confirmed	Not confirmed	% Positive (confirmed)
Overall	200,936	698 (0.3)	200,238 (99.7)	0.3
Age, y, median (IQR)	47 (28–67)	54 (40–65)	47 (28–67)	
Age group, y				
0–14	17,455 (8.7)	3 (0.4)	17,452 (8.8)	0.02
15–34	54,483 (27.3)	121 (18.0)	54,362 (27.3)	0.2
35–49	33,993 (17.0)	164 (24.3)	33,829 (17.0)	0.5
50–65	41,283 (20.7)	228 (33.8)	41,055 (20.6)	0.6
>65	52,400 (26.3)	158 (23.4)	52,242 (26.3)	0.3
Total	199,614	674	198,940	
Missing	1,322 (0.7)	24 (3.4)	1,298 (0.6)	
Sex				
M	108,940 (54.3)	517 (74.1)	108,423 (54.2)	0.5
F	91,822 (45.7)	181 (25.9)	91,641 (45.8)	0.2
Total	200,762	698	200,064	
Missing	174 (0.09)	0	174 (0.09)	
Nationality				
Saudi	146,254 (72.8)	501 (71.8)	145,753 (72.8)	0.3
Non-Saudi	54,682 (27.2)	197 (28.2)	54,485 (27.2)	0.4
Total	200,936	698	200,238	
Missing	0	0	0	
Healthcare personnel				
Yes	9,289 (5.6)	67 (11.2)	9,222 (5.6)	0.7
No	155,825 (94.4)	531 (88.8)	155,294 (94.4)	0.3
Total	165,114	598	164,516	
Missing	35,822 (17.8)	100 (14.3)	35,722 (17.8)	

*Values are no. (%) unless indicated. IQR, interquartile range; MERS-CoV, Middle East respiratory syndrome coronavirus.

Each surveillance year, an average of 66,979 (range 60,659–77,886) suspected case-patients were tested for MERS-CoV. On average, 5,431 (range 2,836–9,154) suspected case-patients were tested monthly during the study period (Figure). The average monthly number of suspected case-patients tested did not change significantly from before (5,654 tests during March 2016–June 2018) to after (4,914 tests during August 2018–February 2019) the case definition change (p = 0.31). Overall, and both before and after the definition change, the average monthly percentage of suspected case-patients testing positive was 0.3%. Peaks in positivity occurred in June 2016 (1.1%), June 2017 (0.9%), and February 2019 (0.9%).

**Figure:**
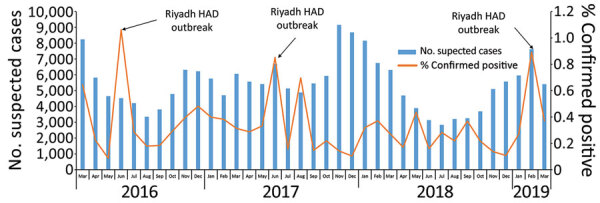
Number of suspected cases and percentage of confirmed positive cases of Middle East respiratory syndrome, Health Electronic Surveillance Network, Saudi Arabia, March 1, 2016–March 20, 2019. HAD, Health Affairs Directorate.

Annually, 203.0 suspected case-patients/100,000 population were tested for MERS-CoV, and 0.7/100,000 population were positive (Table 3). Testing and positivity rates did not vary substantially from year to year (Appendix Table 1). Riyadh HAD had the highest annual testing rate (328.0 suspected cases/100,000 per year). The highest positivity rate per population was in Jouf HAD (2.5 confirmed cases/100,000 population per year) and was largely attributable to an August 2017 outbreak. The study period encompassed 3 Hajj pilgrimage seasons; during these periods, 2,738 pilgrims were tested for MERS-CoV, none of whom tested positive.

**Table 3:** Suspected and confirmed cases of MERS-CoV infection, by local Health Affairs Directorate and among Hajj pilgrims, Health Electronic Surveillance Network, Saudi Arabia, March 1, 2016-March 20, 2019

Local Health Affairs Directorate	Population*	No. confirmed cases/no. suspected cases	% Confirmed positive	No. suspected cases/100,000 population per year	No. confirmed cases/100,000 population per year
Riyadh	8,216,284	340/80,852	0.4	328.0	1.4
Jeddah	4,626,109	37/18,929	0.2	136.4	0.3
Eastern	3,228,261	16/15,856	0.1	163.7	0.2
Makkah	2,319,426	8/13,843	0.06	198.9	0.1
Madinah	2,132,679	25/13,334	0.2	208.4	0.4
Aseer	1,822,189	24/5,560	0.4	101.7	0.4
Jazan	1,567,547	0/1,082	0.0	23.0	0.0
Qaseem	1,423,935	85/9,873	0.9	231.1	2.0
Taif	1,301,778	25/6,342	0.4	162.4	0.6
Ahsa	1,224,600	32/13,009	0.2	354.1	0.9
Tabouk	910,030	9/2,848	0.3	104.3	0.3
Hail	699,774	11/4,946	0.2	235.6	0.5
Najran	582,243	32/2,951	1.1	168.9	1.8
Bahah	476,172	4/2,163	0.2	151.4	0.3
Hafr Al-Baten	447,464	8/681	1.2	50.7	0.6
Bishah	389,686	5/1,131	0.4	112.1	0.4
Nothern	365,231	8/855	0.9	78.0	0.7
Jouf	339,198	25/2,885	0.9	283.5	2.5
Qunfudah	310,453	1/412	0.2	44.2	0.1
Quarayyat	169,277	3/466	0.6	91.8	0.6
Total	32,552,336	698/198,198	0.4	203.0	0.7
Hajj pilgrims	2,352,122	0/2,738	0.0	38.8	0.0

*Health Affairs Directorate and Hajj pilgrim population estimates are from 2017 Saudi Arabia Ministry of Health Annual Statistics Book (*7*). MERS-CoV, Middle East respiratory syndrome coronavirus.

For 82.2% of persons with suspected MERS-CoV infection, a reason for testing was reported (Appendix Table 2). Most were tested because they had signs of pneumonia or acute respiratory distress syndrome (69.9%). Testing because of an epidemiologic link accounted for the highest proportion of positive results overall (0.8%). A higher proportion were tested because of an epidemiologic link after the definition change (19.5%) than before (7.5%).

## Conclusions

Saudi Arabia continues to perform extensive surveillance and testing for MERS-CoV. During the 3-year study period, the MoH tested >65,000 suspected MERS-CoV case-patients per year on average. Of these, 0.3% were positive for MERS-CoV, representing 0.7 confirmed cases/100,000 population per year. As a robust, national surveillance system, HESN enables the geographic and temporal monitoring of trends in testing and surveillance. Compared with HESN MERS-CoV surveillance data from 2015–2016, the percentage of suspected case-patients testing positive (0.7%) and the rate of confirmed cases (1.2/100,000 population) decreased (*8*). Peaks in percentage positivity corresponded to documented MERS-CoV outbreaks (*9*–*11*). The few large recent outbreaks and the reduction in cases might be indicative of robust testing and contact-tracing efforts and early intervention for healthcare infection control.

During the study period, the case definition for suspected cases was revised with the goal of maintaining the sensitivity of the case definition while increasing specificity. Based on limited data (7 complete months of data postrevision), the average number of monthly tests remained constant before and after this change. The change in the case definition is reflected in the reasons for testing persons with suspected MERS-COV infection. A comparison of the reasons for testing before and after the change found that most persons were tested because they had signs of pneumonia or acute respiratory distress syndrome. Unsurprisingly, in both periods, the highest percentage positive was among those with an epidemiologic link to a MERS-CoV patient or exposure to dromedaries.

Comparing data from before and after the case definition change, however, was limited by the relatively short period examined after the definition change and a high percentage of missing data, particularly during implementation of the revised case definition. Although the case definition change took effect in April 2018, HESN was not updated to reflect the change until July 2018. This lag might have affected reporting, and actual implementation of the case definition change likely varied by site. In addition, completeness of the data varied and was likely influenced by differential reporting practices, which affected the availability of data for analysis. Targeted efforts continue to improve the accuracy and completeness of reporting. Additional analysis using more complete data over a longer period would be informative to determine whether the revised case definition might result in changes in testing practices.

Surveillance for MERS-CoV in Saudi Arabia captures and provides important information on suspected and confirmed cases and trends in testing. Continued robust MERS-CoV surveillance is pivotal for the early ascertainment of cases and the effective implementation of control measures.

Appendix Additional information about surveillance and testing for Middle East respiratory syndrome coronavirus, Saudi Arabia, March 2016–March 2019.
